# The Role of Phosphoglycans in the Susceptibility of *Leishmania mexicana* to the Temporin Family of Anti-Microbial Peptides

**DOI:** 10.3390/molecules20022775

**Published:** 2015-02-06

**Authors:** Gabriela A. Eggimann, Kathryn Sweeney, Hannah L. Bolt, Neshat Rozatian, Steven L. Cobb, Paul W. Denny

**Affiliations:** 1 Biophysical Sciences Institute, Department of Chemistry and School of Biological and Biomedical Sciences, Durham University, South Road, Durham DH1 3LE, UK; E-Mails: gabriela.eggimann@durham.ac.uk (G.A.E.); kathrynsweeney@hotmail.com (K.S.); h.l.bolt@durham.ac.uk (H.L.B.); neshat.rozatian@durham.ac.uk (N.R.); 2 School of Medicine, Pharmacy and Health, Durham University, Queen’s Campus, Stockton-on-Tees TS17 6BH, UK

**Keywords:** *Leishmania mexicana*, cutaneous leishmaniasis, drug therapy, antimicrobial peptide

## Abstract

Natural product antimicrobial peptides (AMPs) have been proposed as promising agents against the *Leishmania* species, insect vector borne protozoan parasites causing the neglected tropical disease leishmaniasis. However, recent studies have shown that the mammalian pathogenic amastigote form of *L. mexicana*, a causative agent of cutaneous leishmaniasis, is resistant to the amphibian-derived temporin family of AMPs when compared to the insect stage promastigote form. The mode of resistance is unknown, however the insect and mammalian stages of *Leishmania* possess radically different cell surface coats, with amastigotes displaying low (or zero) quantities of lipophosphoglycan (LPG) and proteophosphoglycan (PPG), macromolecules which form thick a glycocalyx in promastigotes. It has been predicted that negatively charged LPG and PPG influence the sensitivity/resistance of promastigote forms to cationic temporins. Using LPG and PPG mutant *L. mexicana*, and an extended range of temporins, in this study we demonstrated that whilst LPG has little role, PPG is a major factor in promastigote sensitivity to the temporin family of AMPs, possibly due to the conferred anionic charge. Therefore, the lack of PPG seen on the surface of pathogenic amastigote *L. mexicana* may be implicated in their resistance to these peptides.

## 1. Introduction

Leishmaniasis is a neglected tropical disease that is endemic in over 80 countries worldwide. It is caused by *Leishmania* species, insect vector borne protozoan parasites, and affects an estimated 12 million people a year with a further 350 million people living at risk of infection [1]. At the present time a vaccine to prevent leishmaniasis is not available and treatment currently relies entirely on a limited arsenal of chemotherapeutics. For example, treatment of cutaneous leishmaniasis (CL) largely relies on the pentavalent antimonials such as sodium stibogluconate (Pentostam) and meglumine antimoniate (Glucantime) [2,3]. Both Pentostam and Glucantime have been in clinical use for over 70 years despite their associated problems, which include severe side-effects such as cardiotoxicity [4] and the fact that they require parenteral administration [5]. In addition, the use of pentavalent antimonials in the treatment of leishmaniasis is under threat from the emergence of drug resistance [6]. To date resistance has not been widespread in the field but *Leishmania* spp. resistance to Pentostam and Glucantime can be easily induced in the laboratory [7]. Amphotericin B (Fungizone) [8] and diamidine Pentamidine [9] are employed as second-line drugs in the treatment of CL. Like the antimonials, they induce severe side-effects and parasite resistance, although not yet conclusively confirmed in the field, has been observed under laboratory conditions [10]. Given the aforementioned issues with both the current first- and second-line drugs used to treat CL there is clearly a need to develop new and effective therapies for this disease.

In recent years natural product antimicrobial peptides (AMPs) have been investigated as a potential new source of novel antileishmanials [11,12], in part this has been catalysed by the fact that they have displayed promising activity against other cutaneous infectious diseases [13]. The activity of AMPs against *Leishmania* species that give rise to CL has recently been reported. However, despite having promising activity against insect-stage promastigotes, the amphibian-derived temporin family of AMPs has shown limited efficacy against mammalian-stage amastigotes [14]. The predominant surface component of promastigote *Leishmania* is lipophosphoglycan (LPG), a large glycoconjugate which together with cell surface associated proteophosphogycan (PPG) forms a dense glycocalyx protecting the parasite from the mammalian innate immune response on inoculation from the sand fly vector [15]. Subsequently, following infection of macrophage cells and differentiation into the pathogenic amastigote form, expression of cell surface LPG and PPG is massively down-regulated [15,16]. Whilst it has previously been predicted that this thick, negatively charged layer protects the promastigote parasite from cationic AMPs by capturing them and preventing interaction with the cell surface [17], the relative resistance of amastigotes to temporin AMPs suggests the opposite. Herein, we report an extended study of the antileishmanial properties of temporins and the examination of the protective or sensitizing effects of LPG and PPG in *L. mexicana,* a causative agent of CL.

## 2. Results and Discussion

### 2.1. The Antileishmanial Properties of Temporin Antimicrobial Peptides

**Figure 1:**
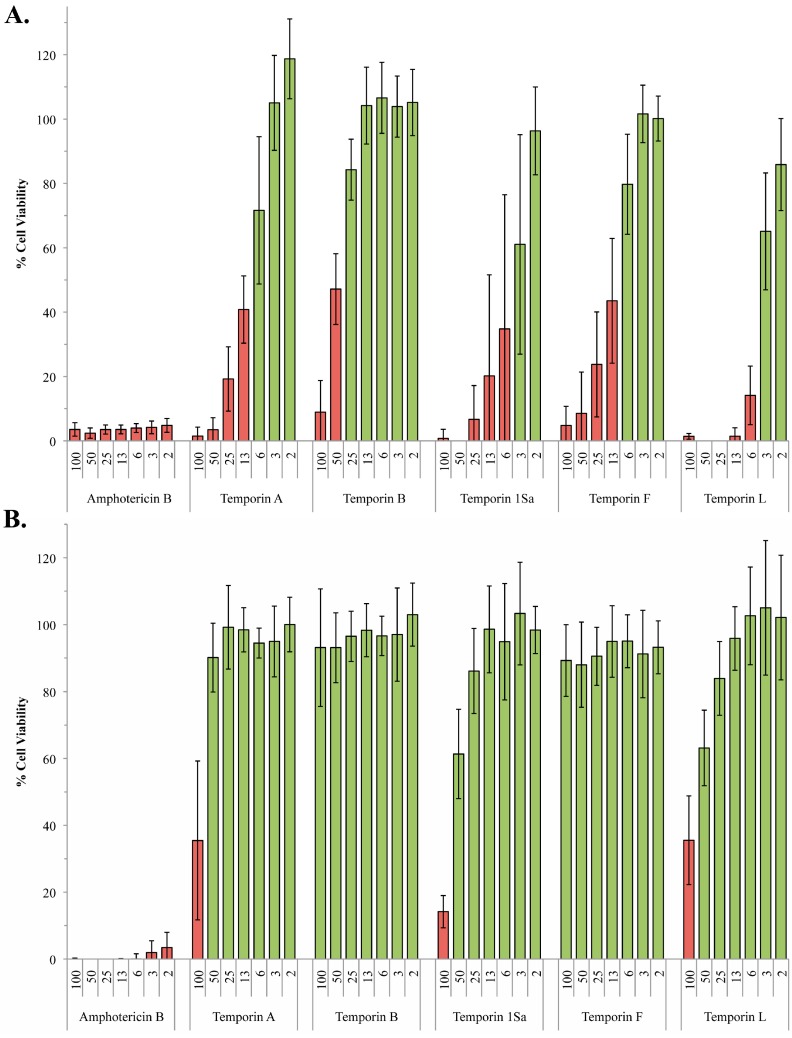
Activity of the temporin peptides against wild type promastigote and amastigote *L. mexicana.* Using the alamarBlue^®^ assay system, *L. mexicana* promastigote (**A**) and amastigote (**B**) viability in the presence of various concentrations (2–100 µM) of the temporin peptides was determined with respect to a DMSO control. Amphotericin B (2–100 µM) was utilized as a positive control. Data points represent the mean of 3 independent experiments performed in triplicate. Standard deviation indicated. Cell viability <50% indicated by red bars.

Temporins A, B, 1Sa, F and L were synthesized and analyzed as described in the Experimental Section. The sequences, formulae and accurate mass data are summarized in Table 1. As previously reported [14] temporin A displayed significant activity against *L. mexicana* promastigotes with an ED_50_ of 8 µM. Whilst temporin B demonstrated low potency with an ED_50_ of 38 µM (Figure 1A; Table 2). However, in contrast to the previous study [14] temporin 1Sa demonstrated good activity against these insect stage forms, an ED_50_ of 4 µM (Figure 1). The reasons for this divergence are not clear, however the previously synthesized 1Sa [14] demonstrated similar activity on reanalysis to that shown in Figure 1, indicating that the assay is likely to be the source of this discrepancy. Notably, serum is observed to mask the efficacy of the temporin peptides [14]. Therefore, it is likely that the serum was inadequately removed before assay in the previous study, thereby leading to the underestimation of 1Sa efficacy. To expand this work further temporins F and L were synthesized and purified as described, and then screened against *L. mexicana* promastigotes. Both AMPs showed good activity, F with an ED_50_ of 14 µM and L with an ED_50_ of 5 µM (Figure 1A; Table 2).

**Table 1:** Tabulated sequences for the peptides tested including accurate mass data. The doubly charged ion was used for accurate mass measurements, *i.e.*, [M+2H]^2+^. All peptides are amidated at the C terminus*.* Lysine (K) and arginine (R) are positively charged side chains.

Peptide	Sequence	Empirical Formula	Mass Calculated [M+2H]^2+^	Accurate Mass Found [M+2H]^2+^
**Temporin A**	FLPLIGRVLSGIL-NH_2_	C_68_H_117_N_17_O_14_	698.9561	698.9548
**Temporin B**	LLPIVGNLLKSLL-NH_2_	C_67_H_122_N_16_O_15_	696.4716	696.4735
**Temporin 1Sa**	FLSGIVGMLGKLF-NH_2_	C_67_H_109_N_15_O_14_S	690.9078	690.9075
**Temporin F**	FLPLIGKVLSGIL-NH_2_	C_68_H_117_N_15_O_14_	684.9531	684.9504
**Temporin L**	FVQWFSKFLGRIL-NH_2_	C_83_H_122_N_20_O_15_	820.9792	820.9792

**Table 2:** ED_50_ for temporins against wild type and mutant *L. mexicana* promastigotes and amastigotes. Mean ED_50_ (and range) shown for the values from at least 3 independent experiments performed in triplicate.

Peptide	ED_50_ (µM)			
	*L. mexicana* Promastiogte	*L. mexicana* Amastigote	*L. mexicana* ∆*lpg1*	*L. mexicana* ∆*lpg2*
**Temporin A**	8 (6–14)	~100	11 (8–16)	26 (21–39)
**Temporin B**	38 (24–64)	>100	39 (28–70)	41 (40–41)
**Temporin 1Sa**	4 (3–13)	42 (35–44)	6 (3–18)	31 (28–35)
**Temporin F**	14 (10–27)	>100	17 (13–29)	23 (16–49)
**Temporin L**	5 (5–6)	83 (46–93)	4 (3–6)	9 (8–12)

However, as previously noted [14], temporin A demonstrated drastically reduced activity against the clinically relevant amastigote form of *L. mexicana* (ED_50_ of approximately 100 µM), whilst temporin B was inactive at the maximal concentration tested, 100 µM. Similarly, temporin F was inactive at 100 µM, whilst 1Sa and L had very low levels of activity against amastigote compared to promastigote forms (ED_50_ 42 and 83 µM respectively; Figure 1B; Table 2).

Temporins A, B and 1Sa have all demonstrated significant activity against axenic amastigotes forms of other *Leishmania* species, A and B against *L. pifanoi* [18] and 1Sa against *L. infantum* [19]. *L. infantum* is an Old World species and a member of the *L. donovani* complex, causing visceral disease in the Mediterranean basin, which has subsequently spread to Latin America where it is sometimes known as *L. chagasi* [20]. Like *L. mexicana*, *L. pifanoi* is a New World species, however it is part of the subgenus *Viannia* whereas *L. mexicana* is part of the subgenus *Leishmania* which actually makes it more closely related to *L. infantum* [21]*.* The considerable evolutionary distance between these different species and subgenra may account for the differing levels of temporin activity observed against amastigotes. The distance is reflected in diversity in the predominant surface glycoconjugates LPG, PPG and glycoinositolphospholipids (GIPLs), macromolecules which play significant roles in the parasite’s interface with its insect and mammalian hosts [22]. However, whilst *L. pifanoi* and *L. infantum* have not been extensively analyzed, the levels of LPG and PPG in amastigotes from both Old and New World species are low or zero [15,16] indicating that other factors confer temperin susceptibility. GIPLs are maintained in both lifecycle stages and although *L. pifanoi* and *L. infantum* remain relatively unstudied, significant inter-species variations in GIPL structure have been elucidated. *L. Viannia panamenensis* decorates the conserved galactose-mannose, glycophosphoinositol core with galactose motifs, whereas *L. donovani* GIPLs are highly mannosylated when compared to the simpler *L. mexicana* structures [22]. It is possible that these significant variations account for the differential activity of some temporin AMPs against axenic amastigotes reported in the literature.

### 2.2. The Role of Phosphoglycans in the Susceptibility of L. Mexiciana to the Temporin Family of Antimicrobial Peptides

**Figure 2:**
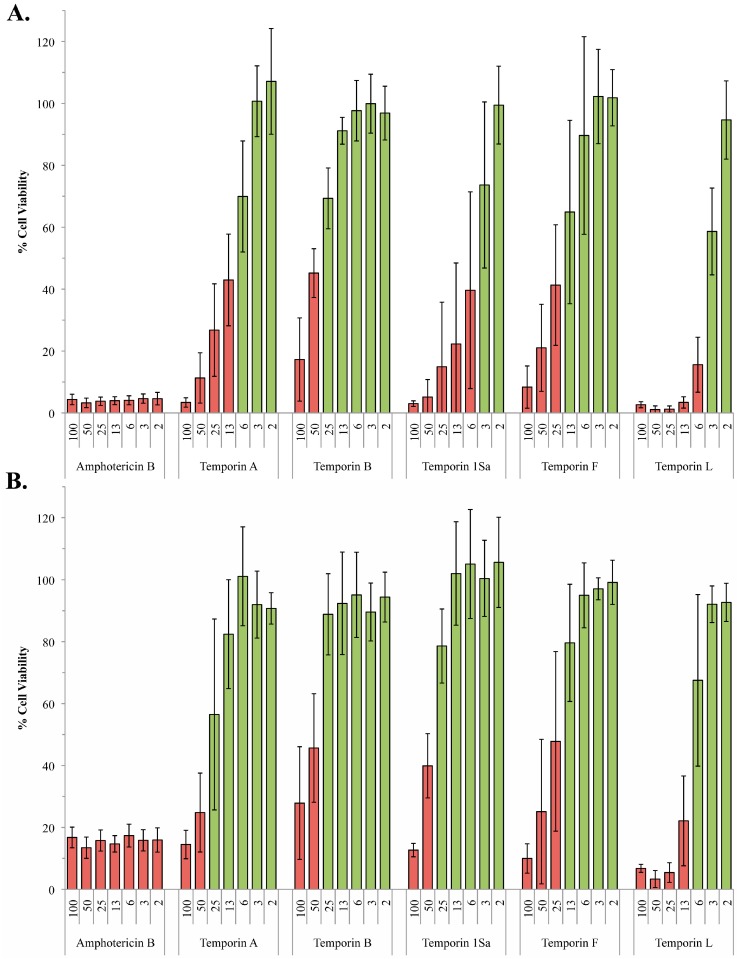
Activity of the temporin peptides against ∆*lpg1 and* ∆*lpg2* promastigote *L. mexicana*. Using the alamarBlue^®^ assay system, *L. mexicana* ∆*lpg1* (**A**) and ∆*lpg2* (**B**) mutant promastigote viability in the presence of various concentrations (2–100 µM) of the temporin peptides was determined with respect to a DMSO control. Amphotericin B (2–100 µM) was utilized as a positive control. Data points represent the mean of 3 independent experiments performed in triplicate. Standard deviation indicated. Cell viability <50% indicated by red bars.

Previously it has been postulated that LPG, which forms a thick negatively charged layer, protects the promatigote parasite from cationic AMPs by capturing them and preventing interaction with the cell surface [17]. However, the fact that amastigote *L. mexicana* possess little or no LPG at their surface [16], and yet are considerably more resistant to the temporin AMPs, argues against this hypothesis. Rather it suggests that LPG is a susceptibility factor, with the negatively charged macromolecule perhaps facilitating the concentration of these cationic peptides at the plasma membrane. To test this hypothesis the five temporins were assayed for their activity against *L. mexicana* promastigotes engineered to lack LPG, ∆*lpg1* [23]. Targeted deletion of the β-galactofuranosyl transferase lpg1 in *L. mexicana* specifically blocked LPG expression, leaving the other phosphoglycans unaffected [23]. The results obtained were perhaps surprising, with the temporins showing little or no difference in efficacy against the ∆*lpg1* mutant when compared with the wild type *L. mexicana* promastigotes (Figure 1A and Figure 2A; Table 2). However, it is notable the that efficacy of temporins A and B have previously been reported to be unchanged against a similar, although less defined, *L. donovani* LPG mutant [18,24].

Proteophosphogylcans (PPGs) are a large range of secreted and cell surface associated parasite glycoconjugates which, like LPG, carry a negative charge [25]. Both axenic and intramacrophage *L. mexicana* amastigotes lack cell surface associated PPG, although the intracellular parasites do secrete a form of PPG known as aPPG [26,27]. Targeted deletion of lpg2, a Golgi GDP-Man transporter, to create ∆*lpg2* [25] prevented the formation of the phosphoglycan repeats that make up the backbone of LPG and PPG leaving the promastigote surface devoid of these negatively charged macromolecules. In contrast to the ∆*lpg1* mutant, the ∆*lpg2* promastigotes appeared less sensitive to all the temporins assayed, with the exception temporin B which was the least active of the AMPs against wild type promastigote forms. Temporins A, 1Sa and L showed the greatest difference in efficacy between wild type and ∆*lpg2* promastigotes (temporin A: ED_50_ 8 µM in wild type *vs.* ED_50_ 26 µM in ∆*lpg2*; 1Sa: 4 *vs.* 31; L: 5 *vs.* 9), with the activity of 1Sa against the mutant close to that of the generically resistant amastigote form (ED_50_ 42 µM in amastigotes *vs.* ED_50_ 31 µM in ∆*lpg2*). This indicated that PPG plays a role in promastigote sensitivity to the temporins (Figure 1 and Figure 2B; Table 2) perhaps due to its negative charge attracting the cationic AMPs to the plasma membrane. However, it is notable that the clinical antileishmanial amphotericin B (used as a control in these experiments) is also less effective against ∆*lpg2 L. mexicana* (Figure 2B) indicating that the lack of PPG is leading to a generic increase in resistance to disruptors of the parasite cell surface. Like the temporins, amphotericin B is a pore forming compound [28].

## 3. Experimental Section

### 3.1. Materials and Reagents

Abbreviations for reagents are as follows: *tert*-butoxycarbonyl (Boc); 9-fluorenylmethoxylcarbonyl (Fmoc); trifluoroacetic acid (TFA); triisopropylsilyl (TIPS); *N,N*-dimethylformamide (DMF); dimethyl sulfoxide (DMSO). Solvents and reagents were purchased from commercial sources and used without further purification unless otherwise noted. Rink amide resin (typical loading level 0.6–0.8 mmol/g) was purchased from Merck4Biosciences (Darmstadt, Germany). DMF was purchased from AGTC Bioproducts (National Diagnostics, Hessle, UK). Piperidine and DIPEA were purchased from Sigma Aldrich (Gillingham, UK), PyBOP from Apollo Scientific (Stockport, UK) and Fmoc-protected amino acids from Novabiochem (Merck, Nottingham, UK). Preparative RP-HPLC was performed with a semi-preparative Perkin Elmer (Waltham, MA, USA) Series 200 lc pump fitted with a 785A UV/Vis detector using a SB-Analytical ODH-S optimal column (250 × 10 mm, 5 µm, Waters Ltd, Elstree, UK); flow rate 2 mL/min. Peptides were characterised by accurate LC-MS (QToF mass spectrometer and an Acquity UPLC from Waters Ltd using an Acquity UPLC BEH C8 1.7 µm (2.1 mm × 50 mm) column (Waters Ltd) with a flow rate of 0.6 mL/min and a linear gradient of 5%–95% of solvent B over 3.8 min (*A* = 0.1% formic acid in H_2_O, *B* = 0.1% formic acid in MeCN). Peptide identities were also confirmed by MALDI-TOF mass spectra analysis (Autoflex II ToF/ToF mass spectrometer Bruker Daltonik GmBH, (Coventry, UK) operating in positive ion mode using an α-cyano-4-hydroxycinnamic acid (CHCA) matrix. Data processing was done with MestReNova Version 8.1.

### 3.2. Peptide Synthesis

Rink Amide AM resin (200–400 mesh, 0.79 mmol/g loading) and all Fmoc-protected amino acids used were purchased from Novabiochem, Merck. PyBOP™ was purchased from CEM (Buckingham, UK). HPLC grade solvents were obtained from Fisher Scientific (Loughborough, UK) and all other reagents from Sigma Aldrich. Side chain protecting groups utilised for the Fmoc amino acids were *t*-butyl for Ser, Pbf for Arg, Boc for Lys and Trt for Asn. Temporins A, B and 1Sa were prepared as previously described [14]. Temporins A, B, 1Sa, F and L were prepared via manual Fmoc SPPS. Peptides were synthesised on a 0.1 mmol scale (127 mg of Rink Amide AM resin). Fmoc amino acids (2 equiv.) were coupled using PyBOP (2 equiv.) and DIPEA (4 equiv.) at RT for 1 h on a shaker (380 RPM, manual procedure). For the first C-terminus amino acid, double coupling was used. Also all amino acids from the 7th position were double coupled to increase the yield. Fmoc deprotection was carried out using piperidine/DMF (20% v/v) for 5 and then 10 min at RT. Final peptide cleavage was achieved using TFA:TIPS:H_2_O 95:5:5 (4 mL) at 25 °C with stirring for a minimum of 4 h. The cleavage cocktail was collected, the solvent removed *in vaccuo*, the crude peptides dissolved in acidified H_2_O (0.1% TFA) and lyophilised. Crude peptides were purified by semi-preparative RP-HPLC, by use of a Perkin Elmer series 200 LC pump, 785A UV/Vis detector, using a 250 mm × 10.0 mm, 5 µm SB analytical column; flow rate = 2 mL/min linear gradient elution 0%–100% B over 60 min (A = 0.1% TFA in 95% H_2_O and 5% CH_3_CN, B = 0.1% TFA in 5% H_2_O and 95% CH_3_CN) at λ = 220 or 250 nm. Relevant fractions were collected, lyophilized, and analysed by LC–MS, MALDI-TOF MS and analytical HPLC. See Supplemental Materials for analytical data (MALDI-TOF MS spectra and analytical HPLC spectra). Temporin A [FLPLIGRVLSGIL-NH_2_], temporin B [LLPIV GNLLKSLL-NH_2_], temporin 1Sa [FLSGIVGMLGKLF-NH_2_], temporin F [FLPLIGKVLSGIL-NH_2_], and temporin L [FVQWFSKFLGRIL-NH_2_].

### 3.3. Antileishmanial Assay

*Leishmania mexicana* (MNYC/BZ/62/M379) wild type and LPG mutant parasites (∆*lpg1* [23] and ∆*lpg2* [25]) were maintained at 26 °C in Schneider’s Drosophila media (Sigma Aldrich) supplemented with heat inactivated foetal bovine sera (15% for promastigotes and mutants, and 20% for amastigotes; Biosera). Promastigotes were transformed into axenic amastigotes by a pH and temperature shift as previously described [29]. Cells were counted using a Neubauer Improved Haemocytometer. Cytotoxicity analyses were performed in 96-well plates (Costar, Corning Inc., Amsterdam, The Netherlands) using alamarBlue^®^ (Life Technologies, Paisley, UK) with some modifications to the published, optimized protocol [14]. Briefly, to mitigate against the effects of serum on the efficacy of the peptides, promastigote and amastigote *L. mexicana* were preincubated (26 °C for promatastigotes; 33 °C for amastigotes) with the temporins (2–100 µM) in 10 µL of serum-free media at 4 × 10^6^ mL^−1^ for 1 h before the addition of 90 µL of complete media. Following incubation at the appropriate temperature for 24 h, 10 µL of alamarBlue^®^ was added to each well and the plates incubated for a further 4 h prior to assessing cell viability using a fluorescent plate reader (Biotek UK, Potton, UK; 560EX nm/600EM nm). All data points were in triplicate, with amphotericin B as positive and DMSO as negative controls. All of the experiments described above were carried out on a minimum of three separate occasions to ensure a robust data set was collected.

## 4. Conclusions

In summary, *L. mexicana* amastigotes are generically resistant to the temporin family of AMPs. This is not attributably to their lack of LPG when compared to the insect stage promastigote form. However, promastigote *L. mexicana* lacking both LPG and the other major negatively charged surface macromolecule PPG are notably more resistant to the temporin AMPs than either wild type or the LPG mutant promastigotes. Given that amastigote *L. mexicana* lack LPG and surface-associated PPG, it may be considered that the cell surface of these mutant promastigotes resembles that of the mammalian pathogenic form [26,27], leading to the hypothesis that PPG is at least partly responsible for the sensitivity of promastigote compared to amastigote *L. mexicana.* Notably, for temporin 1Sa, the ED_50_ against ∆*lpg2 L. mexicana* promastigotes (lacking both of LPG and PPG; 31 µM) was closer to that established for amastigote form (49 µM) than the wild type promastigote form (4 µM; Table 2; Figure 1 and Figure 2). This indicated that, at least for temporin 1Sa, PPG is a major factor in promastigote sensitivity. As hypothesized, this could be due to the highly negatively charged PPG attracting the cationic temporin peptides towards their site of action, the plasma membrane. However, alternative explanations are conceivable. For example, both LPG and cell surface associated PPG are glycophosphoinositol lipid-anchored to the *Leishmania* plasma membrane [30] and their absence may significantly alter membrane fluidity and, perhaps, AMP sensitivity. Whatever the mechanism it is clear that the loss PPG (together with LPG) increases the resistance of *L. mexicana* to a range of temporin AMPs. Given the lack of these predominant macromolecules in pathogenic amastigote forms this may preclude their development as antileishmanials, however secreted PPG associates with *L. major* promastigotes to form a mucin-like coat [31]. Given that intramacrophage (but not axenic) *L. mexicana* amastigotes secrete a form of PPG, aPPG [26,27], it is possible that the presence of this macromolecule within the phagolysosome, and its potential association with the parasite cell surface, will sensitize the pathogen to the temporin AMPs.

## Supplementary Materials

Supplementary materials can be accessed at: http://www.mdpi.com/1420-3049/20/02/2775/s1.
