# Supplementary material for: The Role of Phosphoglycans in the Susceptibility of Leishmania mexicana to the Temporin Family of Anti-Microbial Peptides
Source: Molecules. 2015 Feb 6;20(2):2775–85. doi: 10.3390/molecules20022775 (PMC6272152; doi:10.3390/molecules20022775)
Supplement: Supplementary file 1 [file molecules-20-02775-s001.pdf]

Supporting Information

MALDI-TOF mass spectra and LC-MS data for the peptides are given in Table S1. The MALDI mass spectra for the peptides in Table S1 are given below. Analytical HPLC traces for the peptides in Table S1 are also included underneath the MALDI spectrum for each compound. RP-analytical HPLC performed using Perkin Elmer 200 Series LC pump with a Perkin-Elmer 785A UV-vis detector on an SB Analytical column (ODS-H Optimal), 4.6 mm × 100 mm , 3.5 µm; flow rate = 1 mL/min, loop 20 µL. Gradient: 0%–100% B over 30 min; solvent A: 95% H2O, 5% MeCN, 0.05% TFA; Solvent B: 95% MeCN, 5% H_2O, 0.03% TFA.

Table S1. MALDI and LC-MS data. The singly charged and doubly charged ions and the sodium adducts were used for identification, *i.e.*, $[M+H]^+$, $[M+2H]^{2+}$, $[M+Na]^+$. All peptides are amidated at the C terminus.

Peptide	Mass Calculated	LC-MS Mass Found	MALDI Mass Found	RP-HPLC Retention Time (min)
**Temporin A**	$[M+H]^+$ 1396.9	$[M+H]^+$ 1396.9	$[M+H]^+$ 1396.8	17.3
**Temporin B**	$[M+H]^+$ 1391.9	$[M+H]^+$ 1391.9	$[M+Na]^+$ 1413.7	17.5
**Temporin 1Sa**	$[M+H]^+$ 1380.8	$[M+H]^+$ 1380.8	$[M+H]^+$ 1380.7	17.5
**Temporin F**	$[M+H]^+$ 1368.9	$[M+H]^+$ 1368.9	$[M+Na]^+$ 1390.8	16.6
**Temporin L**	$[M+2H]^{2+}$ 821.0	$[M+2H]^{2+}$ 820.7	$[M+H]^+$ 1640.9	15.9

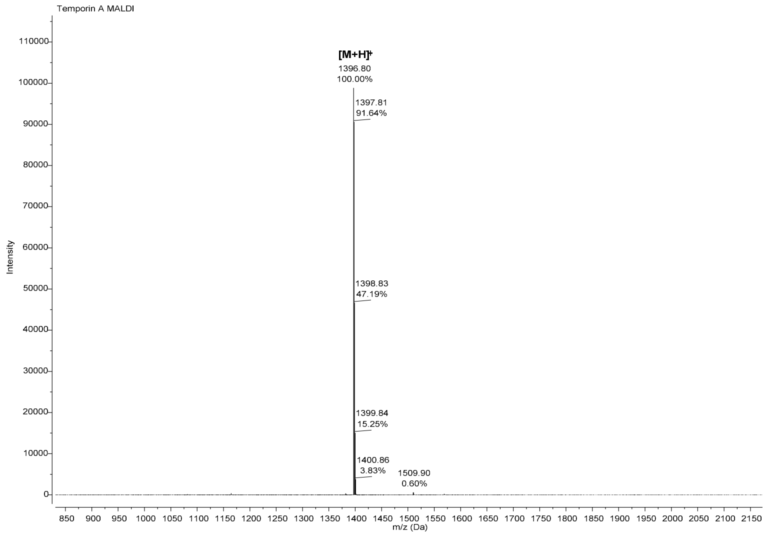

(**A**)

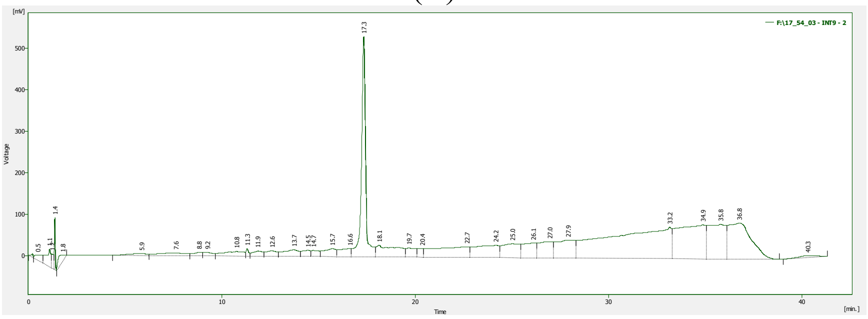

(**B**)

Figure S1. (**A**) Temporin A: FLPLIGRVLSGIL-NH_2; (**B**) Analytical HPLC for Temporin A: FLPLIGRVLSGIL-NH_2.

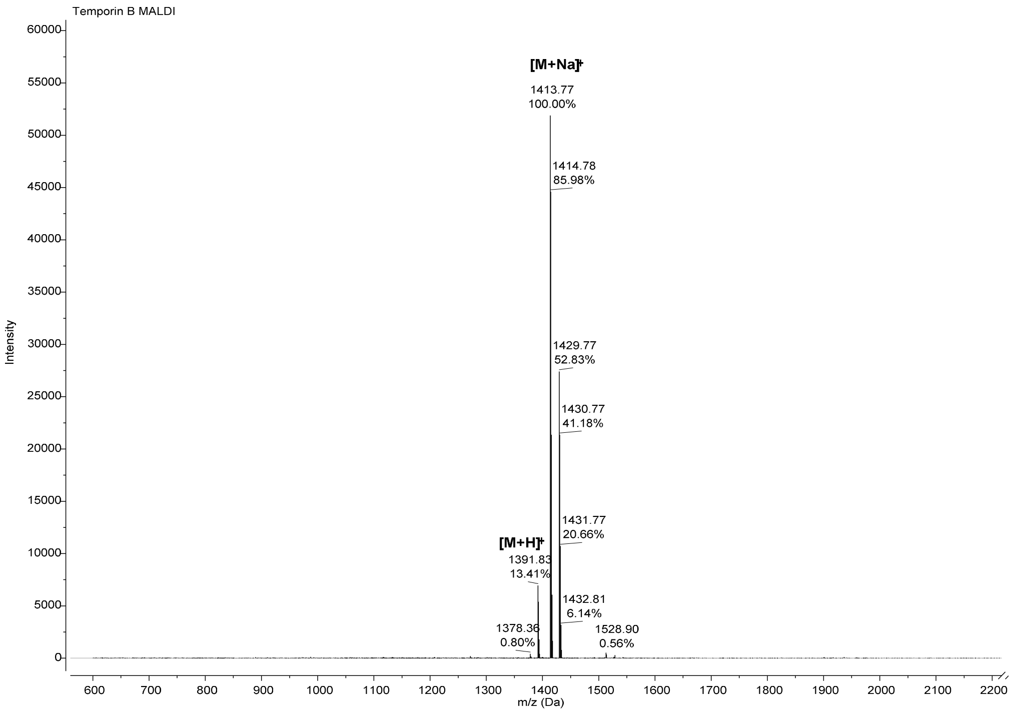

(**A**)

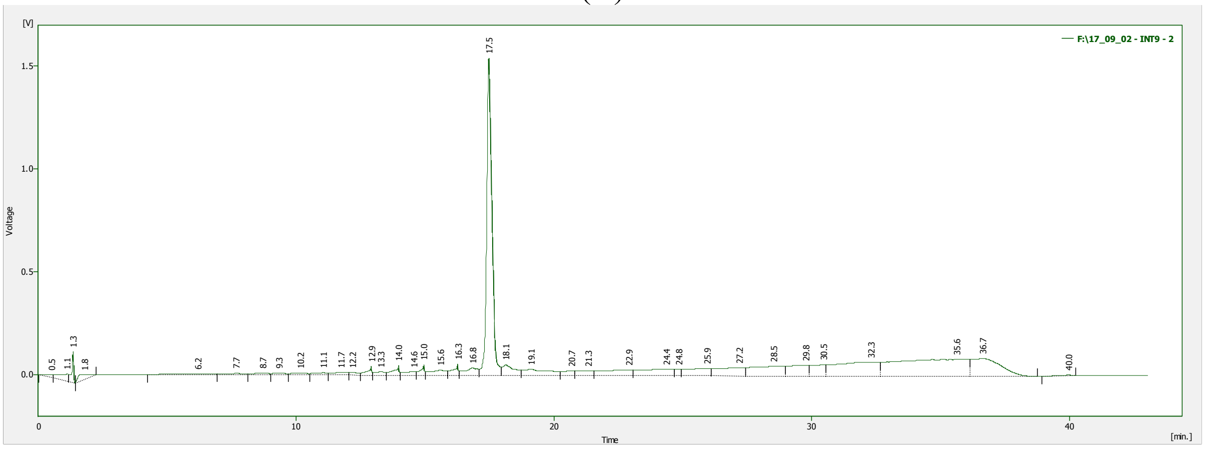

(**B**)

Figure S2. (**A**) Temporin B: LLPIVGNLLKSLL-NH_2; (**B**) Analytical HPLC for Temporin B: LLPIVGNLLKSLL-NH_2.

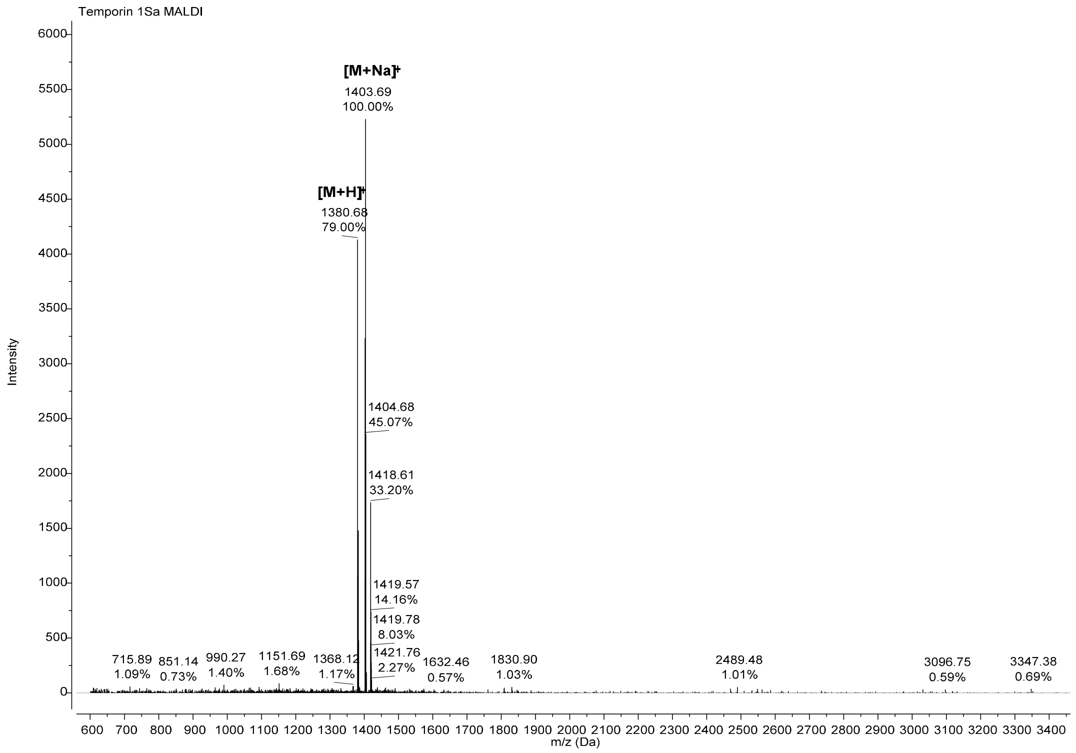

(**A**)

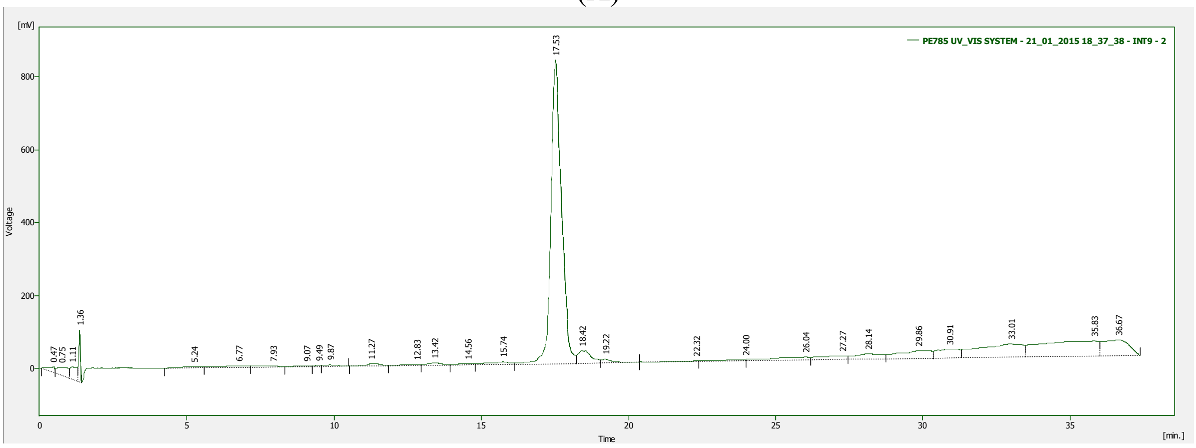

(**B**)

Figure S3. (**A**) Temporin 1Sa: FLSGIVGMLGKLF-NH_2; (**B**) Analytical HPLC for Temporin 1Sa: FLSGIVGMLGKLF-NH_2.

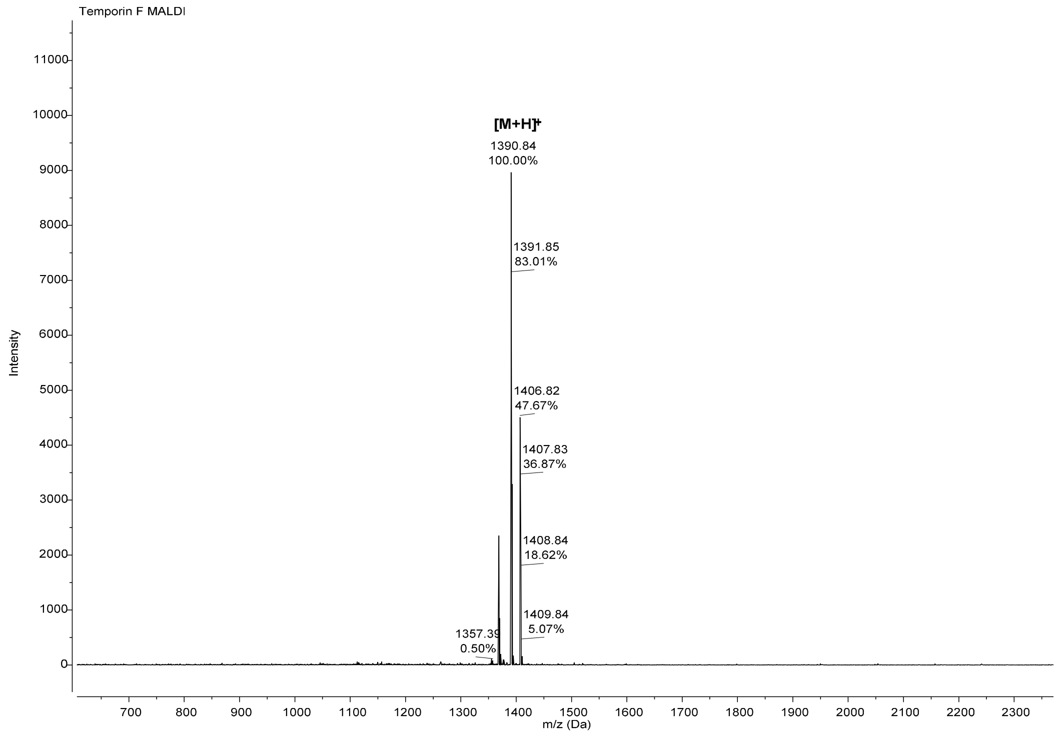

(**A**)

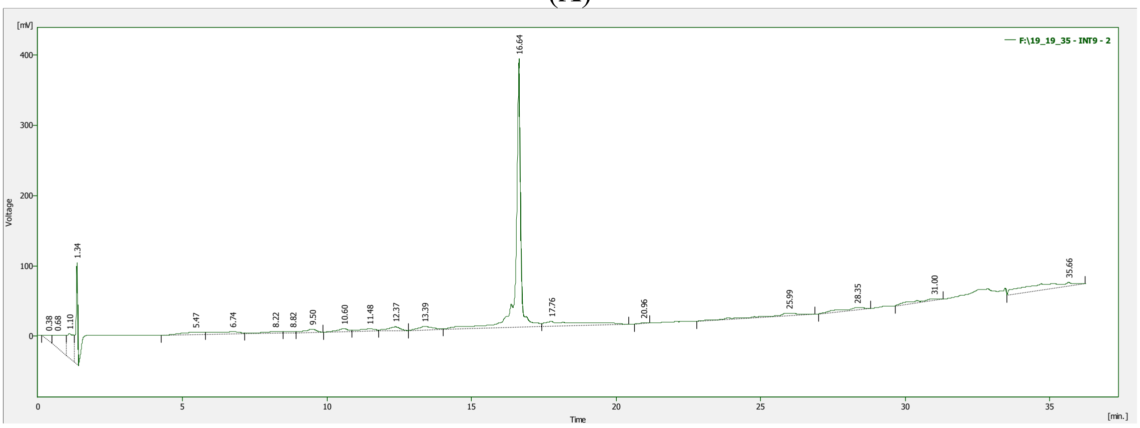

(**B**)

Figure S4. (**A**) Temporin F: FLPLIGKVLSGIL-NH_2; (**B**) Analytical HPLC for Temporin F: FLPLIGKVLSGIL-NH_2.

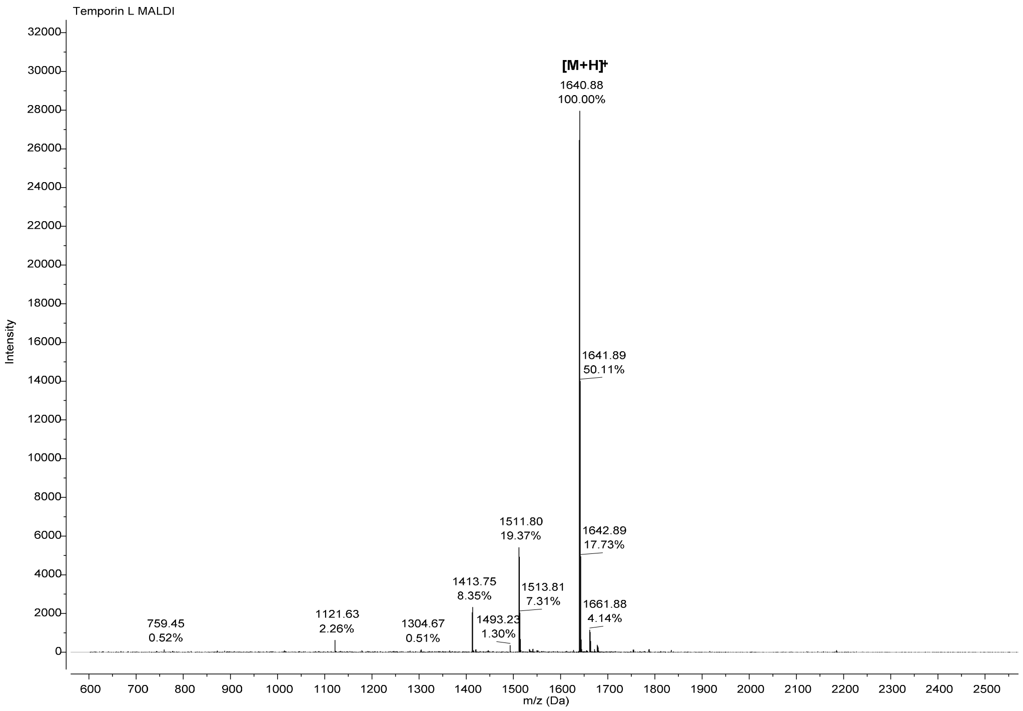

(**A**)

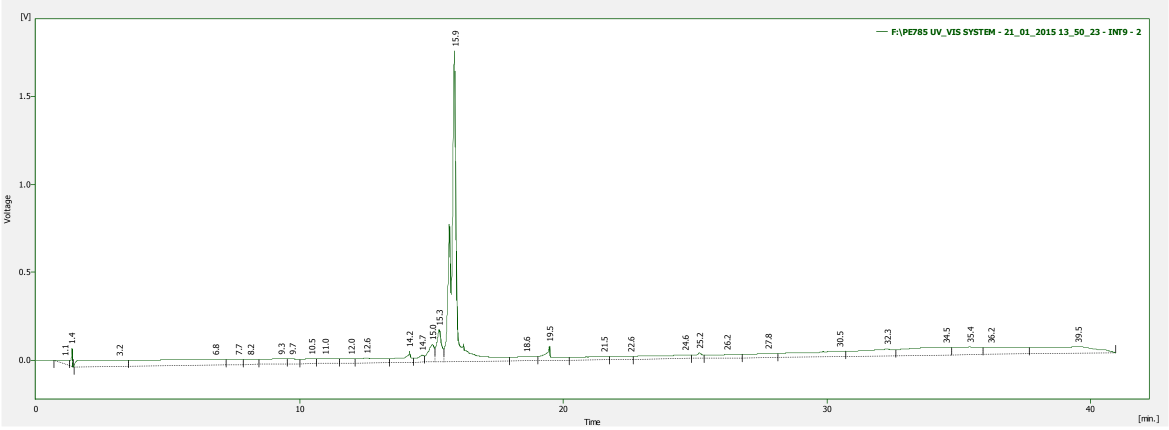

(**B**)

Figure S5. (**A**) Temporin L: FVQWFSKFLGRIL-NH_2; (**B**) Analytical HPLC for Temporin L: FVQWFSKFLGRIL-NH_2.
